# Cardiovascular and Cancer Risk: The Role of Cardio-oncology

**Published:** 2018-03-01

**Authors:** Jessica Shank Coviello

**Affiliations:** Yale School of Nursing and Smilow Cancer Hospital, New Haven, Connecticut

## Abstract

Cardio-oncology is a subspecialty of cardiology. It was created to address oncology data indicating that newly developed drugs for cancer treatment were having unanticipated cardiac side effects. Cardio-oncology designs primary and secondary risk strategies through surveillance as well as interventions to reduce cardiovascular risk (CVR), prevent cardiotoxicities, and manage the side effects that may occur. Rather than discuss in detail the cardiotoxicities of specific therapies or radiation, this review article will explore the interplay of cancer, cancer treatment, and CVR. It will examine the link between CVR and cancer risk, define mechanisms associated with cardiotoxicity, and describe screening and surveillance for patients undergoing cancer treatment. Finally, effective preventative and management strategies used to reduce the incidence of cardiotoxicities in those receiving chemotherapeutics or radiation will be presented.

Heart disease and cancer, although seen as two distinct diseases, have significant commonalities, including risk factors, genetic, metabolic, and inflammatory components, and shared preventative strategies. Together, they represent the most common cause of death in the United States (Curigliano et al., 2016; de Moor et al., 2013; Mehta et al., 2018; Weir et al., 2016; Zamorano et al., 2016).

Previous work has focused on the increasing number of cancer survivors related to improved treatment outcomes (de Moor et al., 2013; Weir et al., 2016). Overall survival and quality of extended survival are key treatment outcomes for the 14.7 million cancer survivors in the US and close to 30 million cancer survivors worldwide (de Moor et al., 2013; Weir et al., 2016).

It is widely accepted that we will continue to see this trend in long-term survival (Curigliano et al., 2016; de Moor et al., 2013; Mehta et al., 2018; Weir et al., 2016; Zamorano et al., 2016). This requires clinicians to adopt a more global view of prevention and seeing both cancer and heart disease as uniquely linked. Such prevention strategies have the potential to successfully improve the quality of life for survivors by reducing both leading causes of death. Cardio-oncology offers a much-needed multidisciplinary approach to optimize a person’s cardiovascular health while realizing the positive outcomes of cancer therapy (Albini et al., 2010; Coviello & Knobf, 2013; Haque et al., 2014; Tromp, Steggnik, Van Veldhuisen, Gietema, & van der Meer, 2017; Yeh, 2011; Zamorano et al., 2016).

## BARRIERS TO CARDIO-ONCOLOGY

There are multiple barriers to reducing or preventing cancer treatment-associated cardiovascular adverse effects: routine screening to identify cardiovascular risk factors (CVRFs) is lacking; clinicians often fail to integrate preexisting CVRFs into treatment planning; standard 2D echocardiography is widely used but has low diagnostic sensitivity to detect cardiac damage; knowledge of the effects of novel targeted agents and checkpoint inhibitors on the cardiovascular system is still emerging; and cardiac biomarkers that may identify subclinical disease are not systematically evaluated (Albini et al., 2010; Barac et al., 2015; Jurcut et al., 2008; Peng, Pentassuglia, & Sawyer, 2010; Wickramasinghe, Nguyen, Watson, Vorobiof, & Yang, 2016). There are no evidence-based standards for cardiovascular risk (CVR) assessment before or after cancer therapy in adult cancer survivors despite a growing body of evidence that they are needed (Abu-Khalaf & Harris, 2009; Armenian et al., 2017; Barac et al., 2015; Daher & Yeh, 2008; Khakoo & Yeh, 2008; Mehta et al., 2018; Patnaik, Byers, DiGuiseppi, Dabelea, Denberg, 2011; Yancy et al., 2013; Zamorano et al., 2016). Identification of patients at risk for cardiovascular disease (CVD) due to personal susceptibility factors and/or cancer treatment would direct risk reduction and therapeutic interventions to reduce morbidity and mortality (Armenian et al., 2017; Barac et al., 2015; Giordano & Hortobagyi, 2007; Granger, 2006; Mehta et al., 2018; Zamorano et al., 2016).

## CARDIOVASCULAR AND CANCER RISK

**Figure 1:**
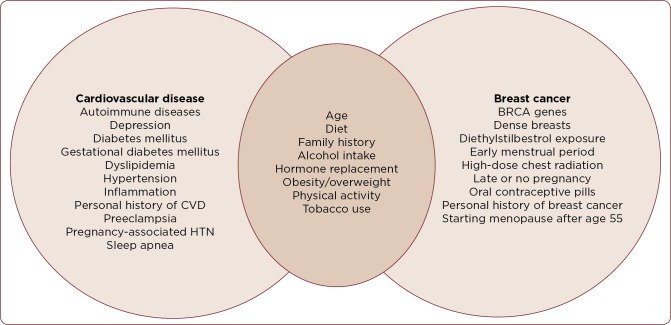
Risk factors for CVD and breast cancer are both shared and separate. CVD = cardiovascular disease; HTN = hypertension. Reprinted with permission from Circulation 2018;137:e30-e66. ©2013 American Heart Association, Inc.

Cardiovascular disease and cancer share similar risk factors, including obesity, sedentary lifestyle, smoking, and chronic inflammation. Recently, Mehta et al. (2018) highlighted those CVRFs that overlap with risk factors for breast cancer (Coviello & Knobf, 2013; see Table 1, Figure 1). Cardiovascular risk factors can be enhanced during cancer treatment and lead to an increased risk of coronary artery disease (CAD) in survivors (Chang, Moudgil, Scarabelli, Okwuosa, & Yeh, 2017a; Chang, Okwuosa, Scarabelli, Moudgil & Yeh, 2017b; Darby et al., 2013; Greenlee et al., 2017; Hague et al., 2014; Marmagkiolis et al., 2016; Weaver et al., 2013; Weaver, Jessup, & Mayer, 2013). Increased CVD morbidity and mortality may be attributed to preexisting CVD in newly diagnosed cancer patients, cardiotoxicity resulting in decreased ventricular function, and treatment effects that result in increased CVRFs such as dyslipidemia, hypertension, central adiposity, and metabolic syndrome (Azard & Denmark-Wahnefried, 2014; Cespedes Feliciano et al., 2016; Coviello, Knobf, & Laclergue, 2013; Gristina et al., 2015; Simon et al., 2018). Genetic factors that produce variable susceptibility to both cancer treatment and toxicity may also be at play (Buzdar, Marcus, Blumenschein, & Smith, 1985; Patel, 2016; Smit, Noordam, le Cessie, Trompet, & Jukema, 2017).

**Table 1:**
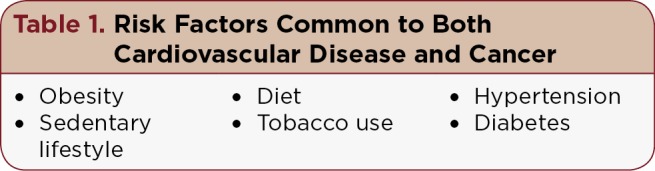
Risk Factors Common to Both Cardiovascular Disease and Cancer

In addition, there is evidence that certain pathophysiologic mechanisms are similar in the etiology of both diseases. Chronic inflammation has been identified as the link in the development of cancer and CVD. Recent studies have shed new light on key pathophysiologic pathways to the development of cancer and CVD. They point to nitric oxide and other free radicals that contribute to both the process of carcinogenesis and the development of microvascular disease (Bellinger et al., 2015; Houben, Martens, & Stehouwer, 2017; Tromp et al. 2017; Wickramasinghe et al., 2016; Zamorano et al., 2016).

The burden of CVR increases in the context of chemotherapy due to increase in weight, central obesity, cholesterol, blood sugar, or blood pressure; however, few studies have looked at pretreatment risk (Barac et al., 2015; Cardinale, Bacchiani, Begggiato, Colombo, & Cipolla, 2013; Coviello & Knobf, 2013; Pituskin et al., 2016). Patients previously treated with chemotherapy and radiation therapy are at increased CVR, with this risk higher than the actual risk of tumor recurrence (Barac et al., 2015; Coviello & Knobf, 2013; Darby et al., 2013; Greenlee et al., 2017; Haque et al., 2014; Marmagkiolis et al., 2016; Mehta & Bairey Merz, 2012; Ward et al., 2012; Weaver et al., 2013). In long-term cancer survivors, a higher incidence of hypertension, dyslipidemia, metabolic syndrome (particularly in breast cancer survivors), acute coronary syndromes, myocardial infarction (MI), and stroke have been reported (Barac et al., 2015; Coviello & Knopf, 2013; Darby et al., 2013; Greenlee et al., 2017; Haque et al., 2014; Marmagkiolis et al., 2016; Weaver et al., 2013).

A 7-fold higher mortality rate, a 15-fold increased rate of heart failure (HF), a 10-fold higher rate of CVD, and a 9-fold higher rate of stroke are seen in cancer survivors compared to the general population. Baseline risk factors and heart disease being equal, patients previously treated with chemotherapy (especially those treated with anthracyclines) have been shown to have an increased risk of cardiomyopathy, HF, and MI in the subsequent 20 years (Barac et al., 2015; Curigliano et al., 2016; de Moor et al., 2013; Mehta et al., 2018; Weir et al., 2016; Zamorano et al., 2016).

For the oncology patient who faces potentially cardiotoxic treatment, clinician awareness of shared risk factors for CVD and cancer is important to prevent added risk burden. In conjunction with oncology, one of the goals of cardio-oncology is to mitigate CVR as patients progress through their cancer treatment.

## LEFT VENTRICULAR DYSFUNCTION: SILENT DANGER

Although there are several cardiovascular side effects that can be seen with potentially cardiotoxic cancer therapy, silent, asymptomatic changes in left ventricular (LV) function represent the greatest cardiovascular concern for patients receiving cancer treatment. Asymptomatic decreases in LV ejection fraction (LVEF) can occur in up to 20% of patients (Armenian et al., 2017; Chang et al., 2017a, 2017b; Suter & Ewer, 2013; Tromp et al., 2017; Yancy et al., 2013; Zamorano et al., 2016). This can happen with both chemotherapy, particularly anthracyclines, and/or radiation therapy to the mediastinum or the left chest. In chemotherapy, the toxicity is a direct result of drug uptake by the cardiomyocyte. Only when the number of myocytes affected reaches a critical threshold does the patient develop symptoms such as shortness of breath or edema. It is during this silent phase of LV dysfunction that monitoring becomes important so that early interventions can be instituted. Risk associated with these insidious changes provides the impetus for a thorough CVR assessment prior to starting high-risk chemotherapy or radiation therapy.

## CLINICAL, GENETIC, METABOLIC, AND INFLAMMATORY FACTORS ASSOCIATED WITH AN INCREASED RISK FOR CARDIOTOXICITIES

Inflammation, cellular metabolic changes, individual genetic susceptibility, and fibrosis represent common causes for loss in LV function during treatment. With anthracyclines, changes occur either during the initiation of the drug or within the year. Acute anthracycline toxicity can appear at or within 1 week of administration. It resembles an acute toxic myocarditis with myocyte damage, inflammatory infiltrates, and interstitial edema. It manifests primarily with electrocardiogram (EKG) changes (20%–30%) and arrhythmias (3%), and may demonstrate some reversible cardiac dysfunction when the drug is held. Much more common is the chronic presentation, with early onset within 1 year or late onset more than 1 year after completion of therapy. This type is marked by cardiac dysfunction rather than EKG changes (Armenian et al., 2017; Cardinale et al., 2015; Chang et al., 2017a, 2017b; Zamorano et al., 2016).

The most important risk factor in anthracycline cardiotoxicity is cumulative dose, with symptomatic LV dysfunction occurring in up to 5% of patients with a cumulative dose of 400 mg/m² (Armenian et al., 2017; Cardinale et al., 2013; Chang et al., 2017a, 2017b; Meinardi et al., 1999; Zamorano et al., 2016). The LV dysfunction in anthracycline therapy is considered type I and is often irreversible (see Table 2).

**Table 2:**
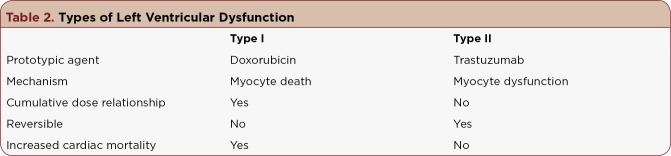
Types of Left Ventricular Dysfunction

Left ventricular dysfunction seen with trastuzumab, an ErbB2 antagonist, can be reversible and is defined as type II LV dysfunction. The adverse effect of trastuzumab is increased significantly in those patients who also receive an anthracycline. Doses are held or reduced if the LVEF drops more than 10% or drops below 50% (Genentech, Inc., 2017).

Acute radiation-induced cardiovascular changes, as seen in pericarditis, are less common today because of newer techniques reducing both irradiated myocardial volume and the total delivered radiation dose (Marmagkiolis et al., 2016; Troost, Thorwarth, & Oyen, 2015). Cardiovascular disease caused by radiation therapy alone is due to inflammation to cardiac tissue that evolves into fibrosis of valves, coronary arteries, the conduction system, and the myocardium.

It has been observed that some patients develop toxicities to both chemotherapy and radiation at low doses while others can tolerate higher doses with no side effects (Buzdar et al., 1985; Patel, 2016; Smit et al., 2017). This variable susceptibility lends itself to the theory that there are genetic and clinical risk factors such as metabolic and inflammatory processes at play. Identifying these would help clinicians develop risk profiles that would aid in identifying underlying pathophysiologic mechanisms as well as identifying interventions to guide treatment (Chang et al., 2017a, 2017b; Patel, 2016; Smit et al., 2017; Zamorano et al., 2016).

## CARDIOVASCULAR RISK FACTORS AND SIDE EFFECTS OF CHEMOTHERAPEUTIC AGENTS

**Figure 2:**
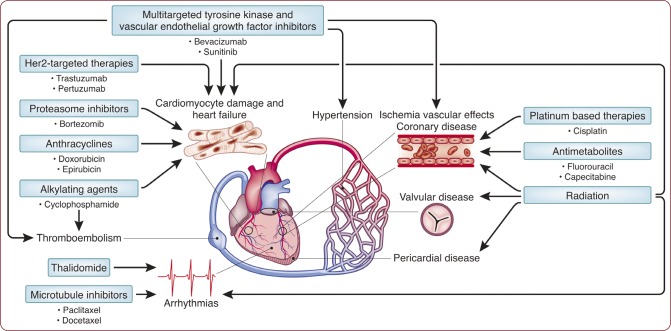
An overview of the cardiovascular side effects of chemotherapy and radiation. HER2 = human epidermal growth factor receptor 2. Reprinted with permission from Circ Res 2016;118:1008-1020. ©2013 American Heart Association, Inc.

Known risk factors for all drug categories include previous CVD or known CVR, age > 65 years or < 5 years, female sex, concurrent radiation, poor nutritional status, genetic susceptibility, poor functional capacity, and low physical activity (Suter & Ewer, 2013; Swain, Whaley & Ewer, 2003; Trompe et al., 2017). Categories of chemotherapeutic agents known to cause cardiovascular side effects can be seen in Table 3. Organ targets associated with cardiotoxicity can be seen in Figure 2.

**Table 3:**
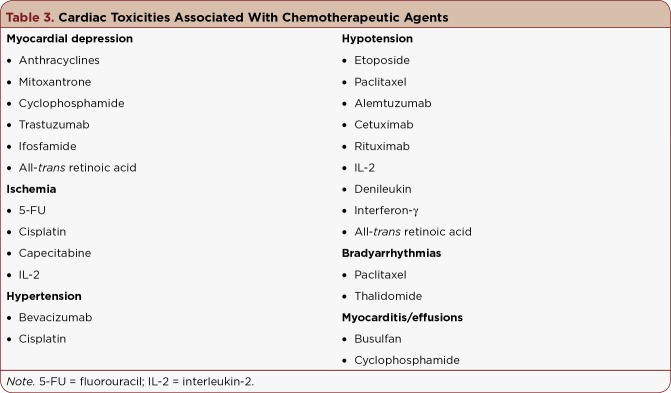
Cardiac Toxicities Associated With Chemotherapeutic Agents

Hypertension is one of the most common CVRFs seen in the general adult population. Its presence as a risk factor is associated with an increase in both anthracycline and trastuzumab LV dysfunction (Barac et al., 2015; Bellinger et al., 2015; Curigliano et al., 2016; Mehta et al., 2018; Tromp et al., 2017; Wickramasinghe et al., 2016; Zamorano et al., 2016). It is also a known causative agent of renal cancer (Colt et al., 2011). Hypertension is seen in tyrosine kinase inhibitors, which target vascular endothelial growth factor receptors causing endothelial dysfunction. There is a high incidence of new hypertension in this drug category (11%–45%). Tyrosine kinase inhibitors are known to destabilize previously controlled hypertension (HTN), accounting for 2% to 20% of severe cases (Izzedine et al., 2009; Milan et al., 2014). Left untreated, LV dysfunction can occur starting as diastolic dysfunction secondary to prolonged increases in afterload. Hypertension is possible from initiation of treatment until 1 year after treatment begins (Barac et al., 2015; Bellinger et al., 2015; Curigliano et al., 2016; Izzedine et al., 2009; Mehta et al., 2018; Tromp et al., 2017; Wickramasinghe et al., 2016; Wu, Chen, Kudelka, Lu, & Zhu, 2008; Zamorano et al., 2016).

## CARDIAC ARRHYTHMIAS

Cardiac arrhythmias and QT prolongation can be seen as a result of a number of different therapies (see Tables Table 4 and Table 5). Preexisting arrhythmias may be seen in 16% to 36% of patients prior to starting therapy (Chang et al., 2017a, 2017b; Curigliano et al., 2016). Providers should anticipate seeing an increase in arrhythmias at baseline as our cancer population ages, increasing the importance of baseline assessment (Lech, 2013; Tamargo, Caballero, & Delpón, 2015; Yeh & Bickford, 2009).

**Table 4:**
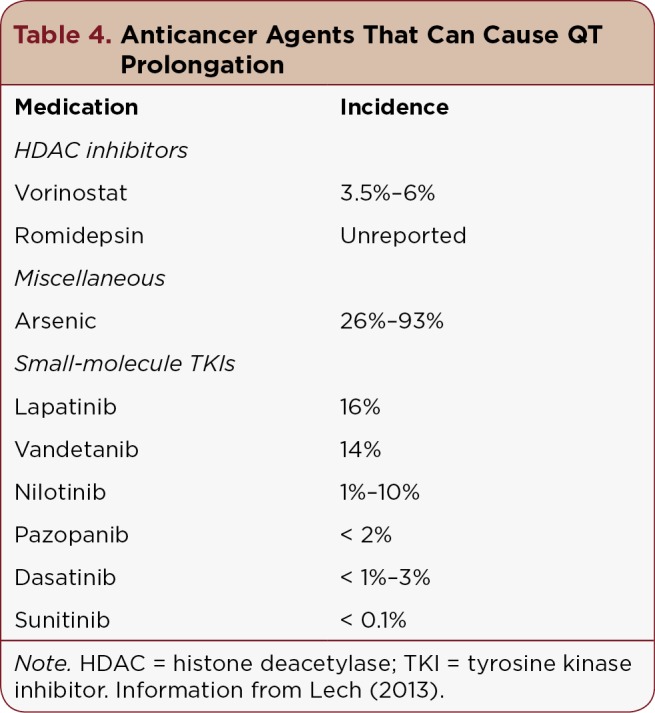
Anticancer Agents That Can Cause QT Prolongation

**Table 5:**
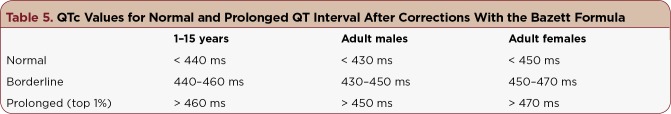
QTc Values for Normal and Prolonged QT Interval After Corrections With the Bazett Formula

Supraventricular arrhythmias are extremely common in the general population with an increase seen in cancer due to tumor, diastolic dysfunction, or atrial enlargement from chemotherapy. The most common is atrial fibrillation, where rate control and stroke prevention need to be considered within the context of the cancer diagnosis and treatment, as well as bleeding risk (Murphy & Salire, 2013).

QT prolongation can be caused by metabolic changes that are a result of fever or dehydration, or renal, liver, or thyroid dysfunction coupled with a chemotherapeutic agent such as arsenic trioxide, which is known to prolong QT in 26% to 93% of patients. The danger of this prolongation is torsade de pointes (polymorphic form of ventricular tachycardia that can lead to cardiac arrest). Changes in treatment are recommended if QTc > 500 ms or if arrhythmias are encountered. Torsade de pointes is rarely seen when QTc is < 500 ms (Lech, 2013; Strevel, Ing, & Siu, 2007; Tamargo, et al., 2015). Frequent EKGs should be used to monitor patients with known CVR, electrolyte disorders, thyroid dysfunction, and in chemotherapy known to prolong QTc. QT measured from the EKG should be corrected using the Bazett or Friderica formula.

Inflammatory responses to checkpoint inhibitors, particularly when used in combination, are due to cytotoxic T-lymphocyte–associated antigen 4 (CTLA-4) and programmed cell death protein 1 (PD-1) regulating immune responses in both cancer and healthy cells. In healthy cells, immunologic checkpoints act to prevent the occurrence of autoimmune reactions. The manipulation of these checkpoints can lead to the occurrence of immune-mediated side effects in any tissue, organ, or system. Side effects may occur weeks or months after the initiation of treatment (Boutros et al., 2016). Although cardiac and pulmonary side effects are seen less often than thyroid, gastrointestinal, pituitary, or dermatologic side effects, myocarditis, pericarditis, Takotsubo-like syndrome, and pneumonitis have been reported (Abdel-Wahab, Shah, & Suarez-Almazor, 2016; Boutros et al., 2016; Larkin et al., 2015; Marrone, Ying, & Naidoo, 2016; Weber, Kähler, & Hauschild, 2012).

## RADIATION THERAPY AND CARDIOTOXICITIES

Radiation therapy is part of treatment in 50% of all malignancies (Baskar, Kuo, Yeo, & Yeoh, 2012; Begg, Stewart, & Vens, 2011; Marmagkiolis et al., 2016). Cardiotoxicity is related to radiation-induced DNA damage, oxidative stress, decreases in nitric oxide metabolism, thrombotic and inflammatory changes resulting in arterial endothelial dysfunction, and myocyte death (apoptosis). These changes are enhanced by traditional CVRFs and in patients who have received anthracyclines (Marmagkiolis et al., 2016). Risk burden leads to a higher incidence of CAD and MI as well as systolic and diastolic dysfunction likely related to this accelerated CAD and myocardial fibrosis (Tromp et al., 2017).

Improved techniques limiting radiation to the cardiac field have reduced the incidence of pericarditis, although it remains a potential side effect, particularly when radiation is combined with certain chemotherapies.

The conduction system can be affected by chest radiation and is likely due to fibrosis. Commonly seen are various degrees of atrioventricular block, right bundle branch block, nonspecific ST changes, low voltage, QT prolongation, supraventricular arrhythmias, and ventricular arrhythmias (Marmagkiolis et al., 2016).

Valvular diseases are known to occur over time, and were more commonly seen when mantle radiation was used in the treatment of Hodgkin lymphoma. The incidence of varying degrees of valvular involvement has been reported as 2% to 37%. Left-sided valves are more commonly affected, with dysfunction due to valvular fibrosis seen 10 to 20 years after completion of treatment (Marmagkiolis et al., 2016).

## EARLY DETECTION OF CARDIAC DYSFUNCTION

Early detection of CVR such as familial predispositions, clinical factors that predispose patients to cardiotoxicity, baseline biomarkers, and imaging can help clinicians determine a plan of care (Barac et al., 2015; Bellinger et al., 2015; Cardinale et al., 2017; Curigliano et al., 2016; Mehta et al., 2018; Tromp et al., 2017; Wickramasinghe et al., 2016; Zamorano, et al., 2016). Cardiac imaging and biomarkers have been found to be useful in baseline assessment as well as the early detection of cardiotoxicity once treatment begins (Altena et al., 2009; Zamorano et al., 2016). N-terminal pro-brain natriuretic peptide (NT-proBNP), troponin I, and brain-type natriuretic peptide (BNP) levels have been shown to predict a drop in LV function during cardiotoxic chemotherapy and can be followed in a serial fashion for continued surveillance (Cardinale et al., 2017; Lenihan et al., 2016; Shah, Yang, Maisel, & Fonarow, 2017).

Although 2D echocardiography is more widely available, 3D echocardiography with myocardial strain has been shown to be more accurate in predicting early LV dysfunction (Armenian et al., 2017; Thavendiranathan et al., 2014; Yancy et al., 2013; Zamorano et al., 2016). Cardiac magnetic resonance (CMR) is useful when there is an added question of cardiac anatomic abnormalities, inflammation, fibrosis, or amyloid (seen with multiple myeloma) or iron deposits, as seen with thalassemia (Tamene, Masri, & Konety, 2015; Yancy et al., 2013; Zamorano et al., 2016). No radiation exposure is associated with either test.

In summary, multiple modalities can be employed to determine risk, as well as to diagnose and treat cardiovascular changes as patients continue cancer therapy. Surveillance strategies are designed based upon preexisting CVR and chronic disease, chemotherapeutic agent used, concomitant radiation therapy, pretreatment imaging, and biomarkers.

## CARDIOVASCULAR RISK AND DISEASE IN THE UNITED STATES

One-quarter of new cancer cases are diagnosed in patients 65 to 74 years of age. The median age at diagnosis is 61 years for breast cancer, 68 years for colon cancer, 70 years for lung cancer, and 66 years for prostate cancer (National Cancer Institute, 2015). With the aging population, we can anticipate that the cardiovascular burden prior to starting cancer therapy will continue to rise.

The major modifiable risk factors for CVD and cancer are well established, overlap, and include obesity, high fat diet, sedentary lifestyle, smoking, and alcohol use. Clinicians inconsistently screen the general public for CVR (Mosca et al., 2007; Mosca et al., 2011; Ng, Chung, Toderika, & Cheng-Lai, 2016). This underscores the importance of a baseline CVR assessment prior to cancer therapy to permit early intervention as well as guide the development of surveillance protocols (Armenian et al., 2017; Chen, 2015; Curigliano et al., 2016; Jacobsen et al., 2016; Zamorano et al., 2016).

## CARDIOVASCULAR RISK IDENTIFICATION AND MANAGEMENT IN SURVIVORSHIP

In addition to the standard risk factors for CVD, chemotherapy and chest radiation need to be considered novel added CVRFs. Risk is additive. During treatment, oncology patients are exposed to a series of cardiovascular insults associated with changes in lifestyle. Frequently, after a diagnosis of cancer, patients stop exercising, tend to increase their body weight, and develop depression, a recognized CVRF. These factors make patients more vulnerable to cardiovascular injuries and increase their risk of premature cardiovascular death. A preexisting CVR is a strong predictor for CVD. Therefore, during the cancer treatment phase, every effort must be made to continue and optimize the therapy of underlying CVD, as well as to correct preexisting and newly acquired CVRs. In addition, as the mechanisms of cardiovascular toxicities in cancer patients differ from the risks of those of the general population, specific guidelines for cancer treatment that take cardiologic conditions into account need to be further developed (Coviello & Knobf, 2013).

## CURRENT BEST PRACTICE GUIDELINES IN THE UNITED STATES

**Figure 3:**
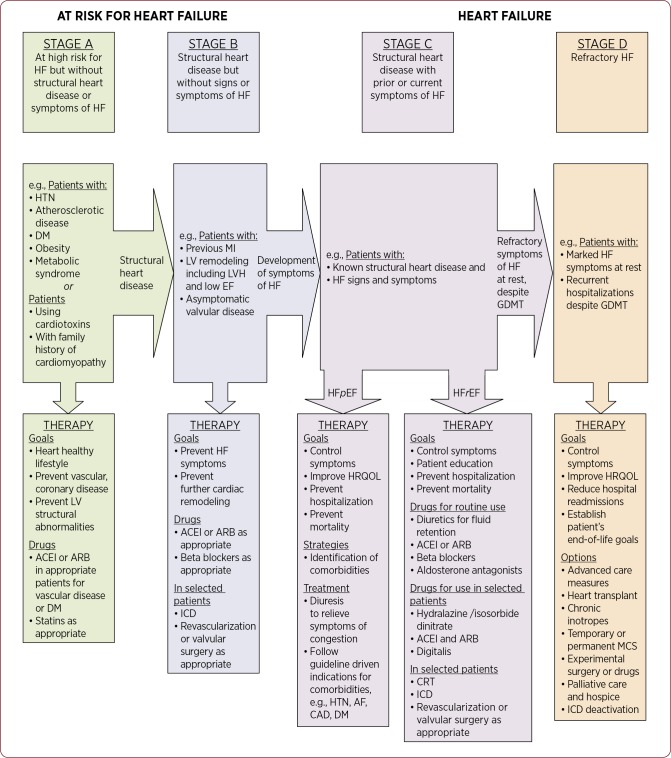
Stages in the development of heart failure and recommended therapy by stage. HF = heart failure; HTN = hypertension; DM = diabetes mellitus; LV = left ventricular; ACEI = angiotensin-converting enzyme inhibitor; ARB = angiotensin-receptor blocker; MI = myocardial infarction; LVH = left ventricular hypertrophy; EF = ejection fraction; ICD = implantable cardioverter-defibrillator; HRQOL = health-related quality of life; AF = atrial fibrillation; CAD = coronary artery disease; GDMT = guideline-directed medical therapy; HFpEF = heart failure with preserved ejection fraction; HFrEF = heart failure with reduced ejection fraction; CRT = cardiac resynchronization therapy; MCS = mechanical circulatory support. Reprinted with permission from Circulation 2013;128;e240-e327. ©2013 American Heart Association, Inc.

The 2013 American College of Cardiology Foundation (ACCF)/American Heart Association (AHA) Guideline for the Management of HF (Yancy et al., 2013), and the Prevention and Monitoring of Cardiac Dysfunction in Survivors of Adult Cancers: American Society of Clinical Oncology Clinical Practice Guideline (Armenian et al., 2017) are based on randomized control trials, but address only LV dysfunction in cancer treatment. Although type I and type II LV dysfunction are specific to cancer treatment, management of LV dysfunction is based on the stages and management of heart failure as outlined by the 2013 ACCF/AHA HF guidelines (see Figure 3).

Cardio-oncology looks at cardiotoxicity through a preventative lens. The focus is aimed at reducing the overall risk of CVD, seeing patients safely through their cancer journey without lasting CVD, and finally, encouraging a lifestyle that reduces further risk of either cancer or CVD. For this reason, cancer patients are considered in Stage A of heart failure—those patients who will receive cancer treatment and are at risk. They are the pretreatment group. In assessing for CVR, the cancer and treatment represent the very first risk factor before other risk factors are assessed. Cancer treatment is in the same category as hypertension in patients who have no structural heart disease. In patients who have demonstrated structural changes during cancer therapy, management is based on Stage B heart failure with known cardioprotective agents (angiotensin-converting enzyme inhibitors and beta or alpha-beta blockers) instituted (see Figure 3).

The recent AHA Scientific Statement, Cardiovascular Disease and Breast Cancer: Where These Entities Intersect (Mehta et al., 2018), and the 2016 European Society of Cardiology Position Paper are more comprehensive but represent expert panel consensus (Mehta et al., 2018; Zamorano et al., 2016). To date, there has not been a large randomized controlled trial that addresses assessment, prevention, and management of CVR in cancer survivors.

## SCREENING AND SURVEILLANCE FOR CARDIOVASCULAR RISK

Screening for CVR is a comprehensive assessment that includes a medical/family history, a specific gynecologic history for women, blood pressure, weight, and waist circumference measurements, and a physical examination. Laboratory tests include EKG, measure of LV function by echocardiogram or CMR, fasting cholesterol, high-density lipoprotein (HDL), low-density lipoprotein (LDL), triglycerides, lipoprotein(a) (Lp[a]), hemoglobin A1c or fasting serum insulin level, glucose, and thyroid function.

Since psychologic distress, commonly measured as anxiety and depression, affects outcomes in men and women with CVD and cancer, screening is strongly recommended as a basis for intervention (Hegel et al., 2008; Szekely et al., 2007). Depression is estimated to occur in 20% to 30% of women with breast cancer (Chen et al., 2010; Institute of Medicine, 2004; Knobf, 2007), and anxiety is prevalent before therapy and at the end of treatment as women transition away from the medical system to survivorship, supporting the need for assessment (Bender, Ergÿn, Rosenzweig, Cohen, & Sereika, 2005; Institute of Medicine, 2004; Norris, Ljubsa, & Hegadoren, 2009).

An EKG is indicated and LV function is assessed in all patients prior to cardiotoxic therapy, but also in patients with long-standing hypertension to detect LV hypertrophy and diastolic dysfunction. The rationale for screening is to identify risk and provide data to target interventions, specifically lifestyle interventions for all risk groups (smoking cessation, healthy eating, regular physical activity, and weight management) and pharmacologic interventions where indicated (Goff et al., 2013; Higgins, O’Halloran, & Chang, 2015; Mehta et al., 2018; Mosca et al., 2007, 2011).

The 2013 American College of Cardiology (ACC)/AHA Pooled Cohort Risk Equation for atherosclerosis cardiovascular disease (ASCVD) is a tool designed to estimate risk in adults aged 21 and older who do not have heart disease or diabetes but who represent a more diverse US population. This score estimates lifetime risk in both African American as well as Caucasian men and women. The tool is easy to use and based on the findings seen in the 2013 Guidelines. This score not only estimates hard (evidence of ischemia or MI) coronary artery disease but also the risk of stroke and peripheral vascular disease. It can be downloaded at tools.acc.org/ASCVD-Risk-Estimator-Plus/#!/calculate/estimate/

The risk factors included in the ASCVD are age, total cholesterol, HDL cholesterol, LDL cholesterol, systolic blood pressure, treatment for hypertension or diabetes, and tobacco use. The calculator also allows you to compare the results to previous estimations. A score of > 7.5% warrants high-dose statin therapy.

Lp(a) is a genetic marker for CVD. Lp(a) excess is the most common inherited lipid disorder in patients with premature CAD and affects about 25% of the population. Studies suggest that Lp(a) is a strong, independent predictor of CVD (Bennet et al., 2009; Clarke et al., 2009; The Emerging Risk Factors Collaboration, 2009).

## BIOMARKERS AS POTENTIAL PREDICTORS OF CARDIOTOXICITY

Two biomarkers, BNP and troponin, have been proposed to identify myocardial injury that will predict subsequent ventricular dysfunction (Barac et al., 2015; Bellinger et al., 2015; Cardinale et al., 2017; Lenneman & Sawyer, 2016; Mehta et al., 2018; Tromp et al., 2017; Wickramasinghe et al., 2016; Yancy et al., 2013; Yu & Ky, 2015; Zamorano et al., 2016). Monitoring biomarkers during cancer treatment may be as important a component to determining overall CVR as monitoring for HTN, diabetes, and obesity, since alone and in combination, their presence may affect left ventricular function and the incidence of HF over time (Table 6).

**Table 6:**
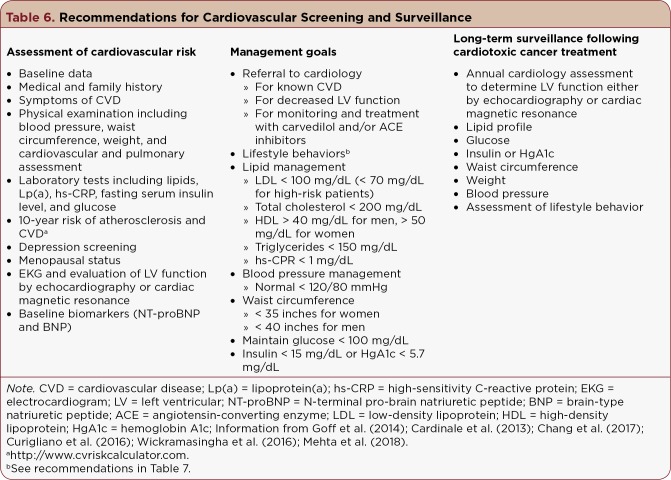
Recommendations for Cardiovascular Screening and Surveillance

## MANAGEMENT

The AHA refers to ideal cardiovascular health as "Life’s Simple 7" (see heart.org/mylifecheck):

- Never smoked or < 1 year
- Body mass index < 25
- Physical activity of 150 min/wk of moderate intensity exercise or 75 min/wk of vigorous intensity exercise
- Total cholesterol < 200 mg/dL
- Blood pressure < 120/80
- Fasting glucose < 100 mg/dL
- 4 of the 5 recommended components of a healthy diet (a diet rich in fruits, vegetables, and fiber, low sodium, low fat, limited intake of processed meats, and increased intake of fish)

The achievement of ideal cardiovascular health is defined as a goal in the Healthy People 2020 national objectives for improving the health of all Americans, set by the US Department of Health and Human Services (see healthypeople.gov; Bambs et al., 2011; Yancy et al., 2013). Many risk factors associated with the development of CVD can be modified with healthy lifestyle interventions. A healthy diet, regular physical activity, smoking cessation, and maintenance of a healthy weight (or weight loss if indicated) can reduce the risk of CVD, cancer, and diabetes (Cardinale et al., 2013; Goff et al., 2013; Mosca et al., 2011; Nayor & Vasan, 2016; Peterson et al., 2017; Whelton et al., 2017).

Exercise as a risk reduction and health promotion strategy in cancer survivors has a strong evidence base (Herrmann et al., 2014; Jones et al., 2016; Pituskin et al., 2016). Exercise in cancer survivors has been shown to improve overall cardiovasular fitness, aerobic capacity, quality of life, psychosocial functioning, muscle strength, and body composition; reduce depression, anxiety, fatigue, and sleep alterations (Courneya et al., 2007; Ferrer, Huedo-Medina, Johnson, Ryan, & Pescatello, 2011; Jones et al., 2011, 2016); as well as decrease the risk of recurrence and improve survival. For cancer survivors who are overweight or obese, weight loss is indicated. Diet plus increased energy expenditure will enhance outcomes. Dietary intervention alone can improve the quality of a survivor’s diet, indicating the adoption of the recommended healthy risk reduction diet (Bail, Meneses, & Demark-Wahnefried, 2016; Coviello & Knobf, 2013; Dunn & Kramer, 2016; Kohler et al., 2016; Pekmezi & Demark-Wahnefried, 2011). Studies suggest that diet combined with cognitive behavioral therapy or counseling can result in weight loss and improved lipids (Duijts, Faber, Oldenburg, van Beurden, & Aaronson, 2011; Thomson et al., 2009). In summary, combined diet and exercise interventions that include components of cognitive behavioral therapy (e.g., motivation, feedback) are indicated to affect behavior changes and achieve goals of weight loss for overweight and obese survivors (see Table 7).

**Table 7:**
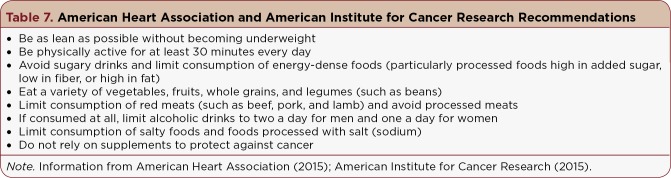
American Heart Association and American Institute for Cancer Research Recommendations

Lifestyle behaviors, specifically physical activity, should be recommended to all survivors to maintain a healthy lipid profile or reduce elevated lipids (Goff et al., 2013; Stone et al., 2014). Pharmacologic therapy is the cornerstone of management for patients with elevated lipids. Statins (3-hydroxy-3-methylglutaryl coenzyme A reductase [HMG-CoA] inhibitors) have established benefit in primary and secondary prevention of acute coronary syndromes, cerebrovascular accident, and venous thromboembolism. Statins targeting lipid metabolism have significant anti-inflammatory properties, and may reduce cardiovascular morbidity and mortality in cancer patients (see Preventing Anthracycline Cardiovascular Toxicity With Statins [PREVENT] trial: clinicaltrials.gov/ct2/show/NCT01988571). Statins are shown to induce apoptosis and inhibit tumor growth, angiogenesis, and metastases along multiple cell lines and may act synergistically with chemotherapy to improve cancer outcomes (Nowakowski et al., 2010; Vaklavas, Chatzizisis, & Tsimberidou, 2011).

## DISCUSSION

Susceptibility to cardiovascular disease in cancer survivors is multifactorial and requires the scientific and clinical knowledge of cardiology and oncology specialists (Chang et al., 2017a, 2017b; Curigliano et al., 2016; Wickramasinghe et al., 2016). It is a critical time for cardiology and oncology to collaborate in risk identification and develop risk reduction interventions if our goal is to enhance the quality of life for cancer survivors and decrease all-cause mortality. Early identification of CVRFs and early referral to cardio-oncology provides the opportunity to target interventions to reduce risk and improve quality of life and survival outcomes. Advanced practitioners are uniquely situated to address this gap in care. Promotion of healthy lifestyle behaviors (physical activity, healthy diet, smoking cessation, and stress management) are important therapeutic strategies to the long-term survival of this vulnerable population (Coviello & Knobf, 2013; Knobf & Coviello, 2011).
